# Quadruple mutation GCAC1809-1812TTCT acts as a biomarker in healthy European HBV carriers

**DOI:** 10.1172/jci.insight.135833

**Published:** 2020-11-19

**Authors:** Kai-Henrik Peiffer, Catrina Spengler, Michael Basic, Bingfu Jiang, Lisa Kuhnhenn, Wiebke Obermann, Tobias Zahn, Mirco Glitscher, Alessandro Loglio, Floriana Facchetti, Gert Carra, Alica Kubesch, Johannes Vermehren, Viola Knop, Christiana Graf, Julia Dietz, Fabian Finkelmeier, Eva Herrmann, Jonel Trebicka, Arnold Grünweller, Stefan Zeuzem, Christoph Sarrazin, Pietro Lampertico, Eberhard Hildt

**Affiliations:** 1 Department of Gastroenterology and Hepatology, University Hospital Frankfurt, Frankfurt, Germany.; 2 Paul Ehrlich Institute, Division of Virology, Langen, Germany.; 3 Institute of Pharmaceutical Chemistry, Philipps-University Marburg, Marburg, Germany.; 4 A.M. and A. Migliavacca Center for Liver Disease, Division of Gastroenterology and Hepatology, Fondazione IRCCS Cà Granda Maggiore Hospital, University of Milan, Milan, Italy.; 5 Department of Medicine, Institute of Biostatistics and Mathematical Modeling, J.W. Goethe University, Frankfurt, Germany.; 6 Department of Gastroenterology, St. Josefs Hospital, Wiesbaden, Germany.; 7 German Center for Infection Research (DZIF), Gießen-Marburg-Langen, Germany.

**Keywords:** Hepatology, Virology, Clinical practice, Molecular biology

## Abstract

Many mutation analyses of the HBV genome have been performed in the search for new prognostic markers. However, the Kozak sequence preceding precore was covered only infrequently in these analyses. In this study, the HBV core promoter/precore region was sequenced in serum samples from European inactive HBV carriers. Quadruple mutation GCAC1809-1812TTCT was found with a high prevalence of 42% in the Kozak sequence preceding precore among all HBV genotypes. GCAC1809-1812TTCT was strongly associated with coexistence of basal core promoter (BCP) double mutation A1762T/G1764A and lower HBV DNA levels. In vitro GCAC1809-1812TTCT lead to drastically diminished synthesis of pregenomic RNA (pgRNA), precore mRNA, core, HBsAg, and HBeAg. Calculation of the pgRNA secondary structure suggests a destabilization of the pgRNA structure by A1762T/G1764A that was compensated by GCAC1809-1812TTCT. In 125 patients with HBV-related cirrhosis, GCAC1809-1812TTCT was not detected. While a strong association of GCAC1809-1812TTCT with inactive carrier status was observed, BCP double mutation was strongly correlated with cirrhosis, but this was only observed in absence of GCAC1809-1812TTCT. In conclusion, our data reveal that GCAC1809-1812TTCT is highly prevalent in inactive carriers and acts as a compensatory mutation for BCP double mutation. GCAC1809-1812TTCT seems to be a biomarker of good prognosis in HBV infection.

## Abstract 2

HBV core promoter/precore region was sequenced in serum samples of European inactive HBV carriers, revealing that GCAC1809-1812TTCT mutation is highly prevalent in inactive carriers.

## Graphical abstract

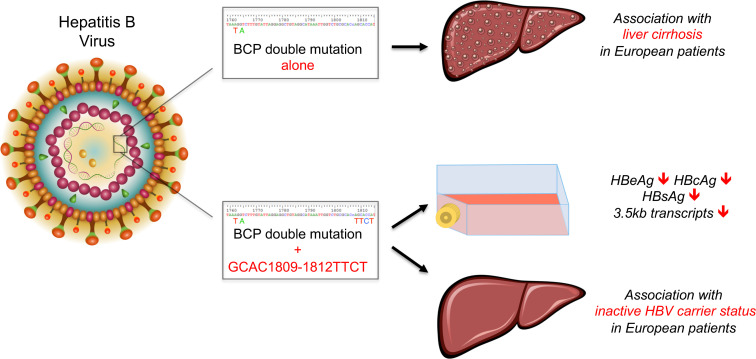

## Introduction

Chronic infection with HBV affects approximately 257 million people worldwide and is a major cause for the development of advanced liver disease and hepatocellular carcinoma (HCC) (1). However, the individual risk for disease progression and/or HCC development is variable and depends on both viral and host factors. Because current antiviral treatment strategies with nucleos(t)ide analogs are usually cost-intensive, long-term therapies with potential side effects, patients who will benefit from this treatment have to be cautiously selected (2–4). Furthermore, patients who do not fulfill treatment criteria have to be followed over a long time period because an increased risk for disease progression and HCC development remains. Therefore, many efforts were made to establish reliable prognostic biomarkers.

In addition to HBV DNA levels and quantitative surface antigen (qHBsAg) levels as established biomarkers (5–9), several viral polymorphisms and mutations in preS gene, precore gene, and basal core promoter (BCP), were extensively studied and found to be associated with the course of disease and treatment response (10–12). However, although described as prognostic markers, they have not been established in daily clinical practice so far. For example, the double mutation A1762T/G1764A is the most common mutation in BCP and was found in some studies to be associated with progression to advanced liver disease and HCC development (11, 13–15). However, although a pronounced prevalence of A1762T/G1764A was observed in patients with advanced liver disease, the frequency of this mutation is also approximately 50% in our cohort (16) of HBV inactive carriers. These data are contradictory and further limit the specificity of A1762T/G1764A as a robust prognostic marker in clinical practice.

The present study aimed to further specify the A1762T/G1764A-related clinical phenotype and to establish additional biomarkers for HBV-related prognosis. We analyzed the genetic variability of the core promoter and the Kozak sequence preceding precore in serum samples of inactive carriers and patients with established compensated HBV-related liver cirrhosis. In addition, we performed bioinformatical modeling of pregenomic RNA secondary structures and in vitro analyses of A1762T/G1764A and the quadruple point mutation GCAC1809-1812TTCT in hepatoma cells.

## Results

### GCAC1809-1812TTCT was highly prevalent in inactive carriers and strongly associated with BCP double mutation A1762T/G1764A.

**Figure 1:**
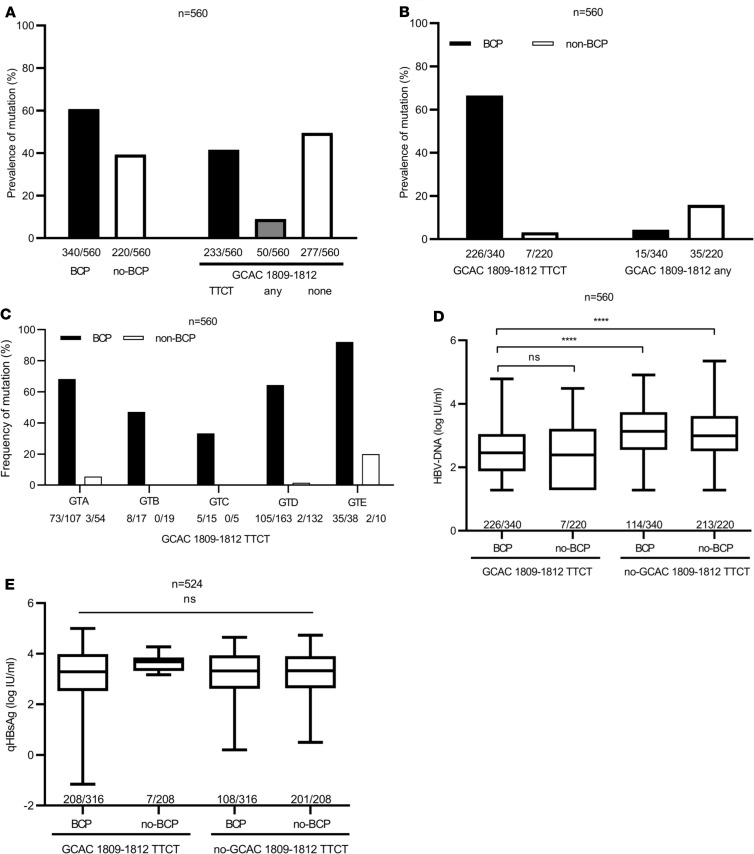
High prevalence of GCAC1809-1812TTCT and association with BCP double mutation A1762T/G1764A and lower HBV DNA levels in HBsAg carriers. (**A–C**) Prevalence of (**A**) BCP double mutation A1762T/G1764A and mutations at nt1809–1812, (**B**) mutations at nt1809–1812 dependent on coexistence of A1762T/G1764A, and (**C**) mutations at nt1809–1812 dependent on coexistence of A1762T/G1764A among different GTs (GTA–GTE) in sera of 560 patients with a chronic HBV infection (HBsAg carriers) from the Albatros cohort. (**D** and **E**) Association of A1762T/G1764A and GCAC1809-1812TTCT with (**D**) HBV DNA levels and (**E**) qHBsAg levels in inactive carriers from the Albatros cohort. Data are shown as follows: median (line inside the box); first and third quartile (upper and lower limit of the box, respectively); and the highest and lowest values are represented by the top and bottom whiskers. A Kruskal-Willis test with a post hoc Dunn’s test were performed to determine statistical significance. *P < 0.05, **P < 0.01, ***P < 0.001, ****P < 0.0001. BCP, basal core promoter; HBsAg, HBV surface antigen; GTs, genotypes.

To search for additional polymorphisms/mutations associated with the clinical phenotype of BCP, double mutation reanalysis of 504 primary sequences (16) and sequencing of additional 56 serum samples of the BCP region of HBsAg carriers from the Albatros study was performed (patients demographics in Table 1). Besides a high prevalence of A1762T/G1764A in 61% (340 of 560) of the samples, we observed that additional mutations in the Kozak sequence at position nt1809–1812 directly preceding the precore start codon were found in 51% (283 of 560) of the samples (Figure 1A). In 42% (233 of 560) of the samples, a quadruple substitution of TTCT instead of GCAC (GCAC1809-1812TTCT) was identified at this position. In addition, in 9% (50 of 560) of the samples, non-TTCT mutations (single, double, or triple point mutations) at position nt1809–1812 were detected (Figure 1A). The presence of GCAC1809-1812TTCT was strongly associated with the coexistence of BCP double mutation A1762T/G1764A (Figure 1B). Although this mutation was found in 66% (226 of 340) of the samples with A1762T/G1764A, in samples without A1762T/G1764A, the GCAC1809-1812TTCT variant was detected only in 3% (7 of 220) of the samples (Figure 1B). In contrast, non-TTCT mutations at position nt1809–1812 were not associated with the BCP double mutation A1762T/G1764A (Figure 1B).

### Association of GCAC1809-1812TTCT with BCP double mutation was independent of the HBV genotype and GCAC1809-1812TTCT was associated with lower HBV DNA levels.

To evaluate a possible genotype dependency, the prevalence of GCAC1809-1812TTCT among HBV genotypes (GTs) GTA–GTE was analyzed. GCAC1809-1812TTCT was frequently detected among all analyzed GTs, with an overall prevalence of 47% (76 of 161) in GTA, 22% (8 of 36) in GTB, 25% (5 of 20) in GTC, 36% (107 of 295) in GTD, and 77% (37 of 48) in GTE. In the BCP-positive subgroup, the GCAC1809-1812TTCT prevalence was higher among all GTs (Figure 1C). Again, a strong association of GCAC1809-1812TTCT with BCP double mutation was found among all GTs.

To analyze the impact of GCAC1809-1812TTCT on the clinical phenotype, the mutation was correlated with HBV DNA and qHBsAg levels. GCAC1809-1812TTCT in combination with BCP double mutation A1762T/G1764A was associated with significantly lower HBV DNA levels (2.51 log IU/mL vs. 3.14 log IU/mL; *P* < 0.001) in comparison with A1762T/G1764A without GCAC1809-1812TTCT and in comparison with samples without any of these mutations (Figure 1D). This association was observed for the overall study population as well as GTA and GTD individually (for detailed analyses in the GT, see Supplemental Figure 1, A–E; supplemental material available online with this article; https://doi.org/10.1172/jci.insight.135833DS1). In contrast, A1762T/G1764A alone without GCAC1809-1812TTCT was not associated with changes in HBV DNA levels. None of these mutations were associated with changes in qHBsAg levels in the overall study population and among the GTs (Figure 1E and Supplemental Figure 2, A–E).

### GCAC1809-1812TTCT reduced HBsAg expression in vitro.

**Figure 2:**
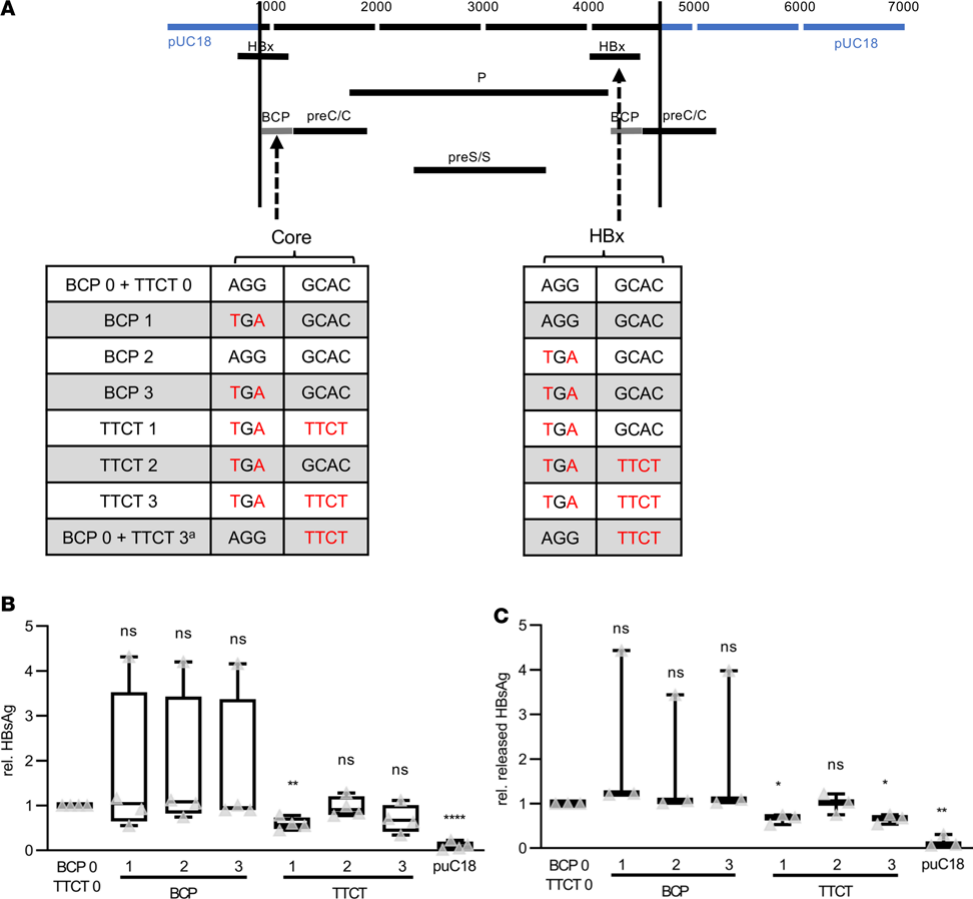
Diminished synthesis of HBsAg by GCAC1809-1812TTCT in vitro. (**A**) Overview of expressed genotype A genomes based on a 1.2-mer isolate from a patient of the Albatros cohort (TTCT3) with both the A1762T/G1764A (BCP) double mutation and the GCAC1809-1812TTCT (TTCT) quadruple mutation in core promoter and HBx (due to partially overlapping reading frame of HBV). 0 = absence of the mutation, 1 = mutation only in core promoter, 2 = mutation only in HBx, and 3 = mutation in both core promoter and HBx. With respect to the clinical data, all variants (TTCT1–3) contain the additional A1762T/G1764A BCP mutation. A construct without these 2 mutations (BCP0 plus TTCT0) was used as a reference. ^a^For analysis of extracellular DNA and HBeAg, an additional genome harboring GCAC1809-1812TTCT in HBx and core promoter but without the A1762T/G1764A BCP double mutation was used (BCP0/TTCT3). A 1.1-mer HBeAg WT genome and an HBeAg-negative genome harboring a G1896A/G1899A precore mutation were used as controls. (**B** and **C**) HBsAg-specific ELISA of (**B**) lysates, *n* = 4, and (**C**) supernatants, *n* = 3. Data are shown as follows: median (line inside the box), first and third quartile (upper and lower limit of the box, respectively) and the highest and lowest values are represented by the top and bottom whiskers. Multiple *t* test with the Holm-Šidák method was performed to correct for multiple group comparisons and to determine statistical significance. *P < 0.05, **P < 0.01, ***P < 0.001, ****P < 0.0001. HBsAg, HBV surface antigen; BCP, basal core promoter.

To further investigate the underlying mechanism that might trigger the correlation of GCAC1809-1812TTCT with the BCP double mutation and lower HBV DNA levels, in vitro experiments were performed. An HBV genome was isolated from a serum sample of 1 patient infected with HBV GTA harboring both A1762T/G1764A and GCAC1809-1812TTCT and this genome was cloned as a 1.2-mer into pUC vector. Based on this genome, a total of 8 vectors containing different combinations of BCP double mutation (BCP) and GCAC1809-1812TTCT mutation (TTCT) were synthesized via site-directed mutagenesis (for an overview of the synthesized vectors, see Figure 2A). Because of the partially overlapping open reading frames of HBV, both of these mutations appear in the core promoter as well as the HBx gene. Therefore, we introduced the mutated or WT-specific sequences in the respective core promoter gene (variant 1), the HBx gene (variant 2), or both genes (variant 3) independently. With respect to our clinical data, the variants harboring the GCAC1809-1812TTCT mutation (TTCT1–3) contain the additional A1762T/G1764A mutation. A construct without these 2 mutations (BCP0 plus TTCT0) was used as a reference.

In hepatoma cells expressing the 2 variants harboring GCAC1809-1812TTCT in the core promoter position (TTCT1 and TTCT3), slightly lower HBsAg levels were detected by HBsAg-specific ELISA (Figure 2B). In addition, HBsAg levels were slightly lower in the supernatants of cells expressing these 2 variants (Figure 2C). In contrast no changes in HBsAg expression were observed in cells expressing the genome with GCAC1809-1812TTCT only in the HBx position (TTCT2). In cells expressing the variants with BCP double mutation without GCAC1809-1812TTCT (BCP1–3), no significant changes in HBsAg expression were observed in the ELISA of lysates and supernatants. These data indicate that GCAC1809-1812TTCT leads to a slight reduction of HBsAg when present in the core promoter.

### GCAC1809-1812TTCT reduced core expression and HBeAg release in vitro.

**Figure 3:**
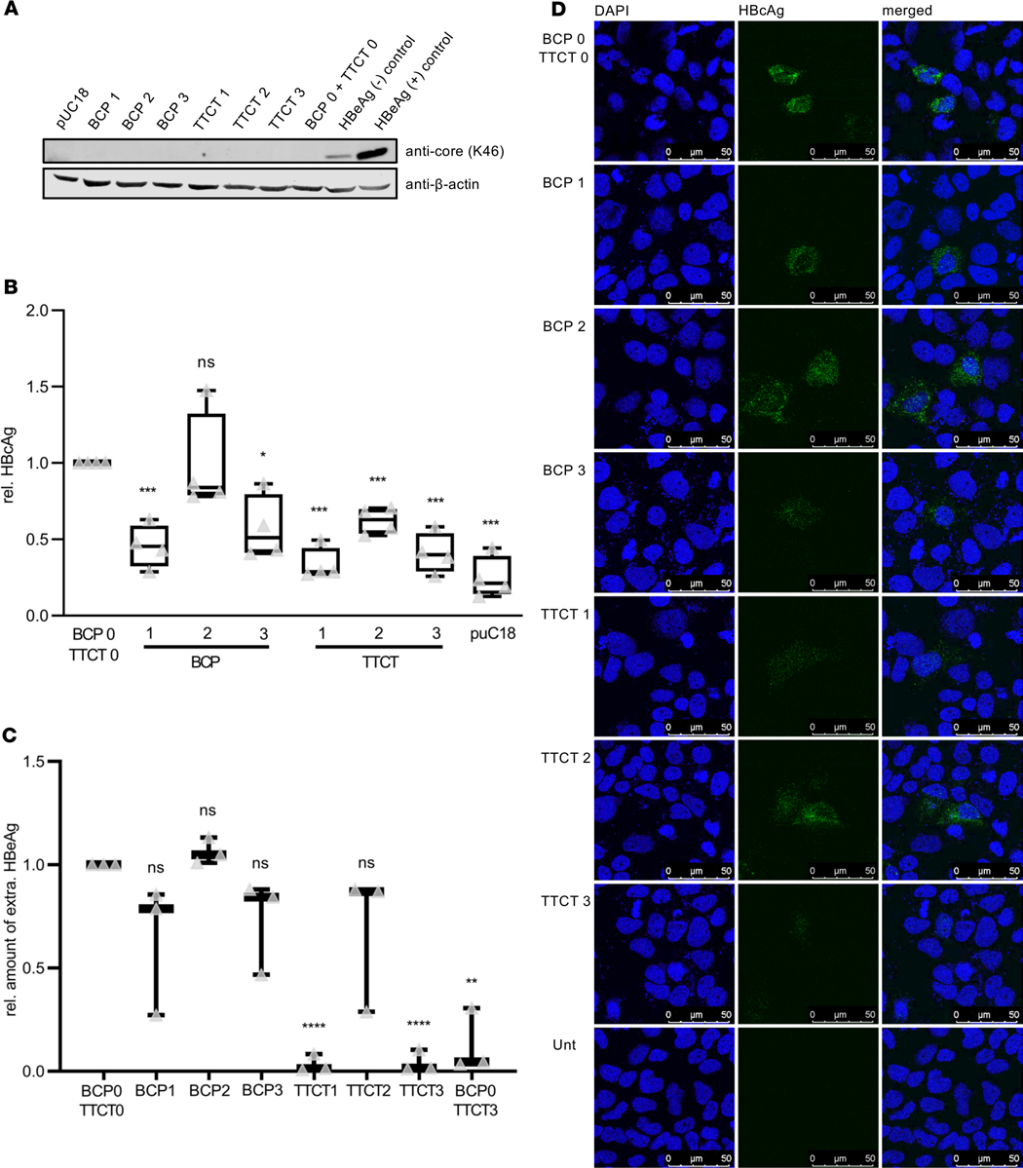
Diminished synthesis of core protein and HBeAg by GCAC1809-1812TTCT in vitro. (**A**) Western blot analysis using a core-specific antibody of lysates; as additional control, a different HBeAg-negative genotype A genome harboring precore double mutation G1896A/G1899A was used. (**B** and **C**) HBcAg- and HBeAg-specific ELISA of lysates and supernatants (*n* = 4 and *n* = 3, respectively). Data are shown as follows: median (line inside the box); first and third quartile (upper and lower limit of the box, respectively); and the highest and lowest values are represented by the top and bottom whiskers. Multiple *t* test with the Holm-Šidák method was performed to correct for multiple group comparisons and to determine statistical significance. *P < 0.05, **P < 0.01, ***P < 0.001, ****P < 0.0001. (**D**) CLSM analysis of transfected Huh 7 cells stained with the core-specific antibody MAB3120. HBsAg, HBV surface antigen.

For core quantification, Western blot, HBcAg-specific ELISA, and immunofluorescence analyses were performed. Western blot analysis using a polyclonal HBcAg/HBeAg-specific serum revealed that almost no core/HBeAg was detected in lysates of cells, which express the variants. The specificity of the blot was proven by using an additional genome containing the precore double mutation G1896A/G1899A, which was used as an additional HBeAg negative control, and by using an HBeAg positive WT genome as a positive control (Figure 3A). In supernatants, core/HBeAg was detected only in case of the HBeAg-positive control via Western blot analysis (data not shown). A core-specific ELISA of lysates detected a significant reduction of core in the cases of the variants harboring BCP double mutation in the promoter position (BCP1 and BCP3 and all TTCT variants, 1–3). However, while the addition of GCAC1809-1812TTCT in the core promoter position (TTCT1 and TTCT3) led to an even stronger reduction of core, GCAC1809-1812TTCT in the HBx position (TTCT2) did not lead to a further reduction of core (Figure 3B). No significant changes in core amount were observed with the variant containing the BCP double mutation in the HBx position (BCP2). The core-specific ELISA of supernatants revealed that the core was not sufficiently detectable (data not shown). In immunofluorescence analysis using an antibody for assembled core, specific but only weak core signals were observed in cells expressing these variants (Figure 3D). In line with the core-specific ELISA, slightly lower core signals were obtained for the constructs with the BCP double mutation in the promoter position (BCP1 and BCP3), and even lower signals were observed in cells expressing the constructs with the additional GCAC1809-1812TTCT mutation in the promoter position (TTCT1 and TTCT3).

For analysis of HBeAg, release semiquantitative HBeAg-ELISA of supernatants was performed (Figure 3C). Here, we observed that HBeAg levels were moderately but not significantly decreased in cells expressing genomes with the BCP double mutation A1762T/G1764A located in the promoter region (BCP1 and BCP3). In contrast, a very strong reduction of HBeAg levels nearly to the detection limit was detected in cells expressing the constructs containing the additional GCAC1809-1812TTCT mutation, but again only when this mutation was present in the core promoter position (TTCT1 and TTCT3). To analyze if the GCAC1809-1812TTCT mutant alone also abolishes the release of HBeAg, we added another construct in this analysis with GCAC1809-1812TTCT in the promoter and HBx position but without the additional BCP double mutation (BCP0 plus TTCT3). Also with this construct, a very strong but slightly less pronounced reduction of HBeAg was observed.

### GCAC1809-1812TTCT impaired RNA synthesis in vitro.

**Figure 4:**
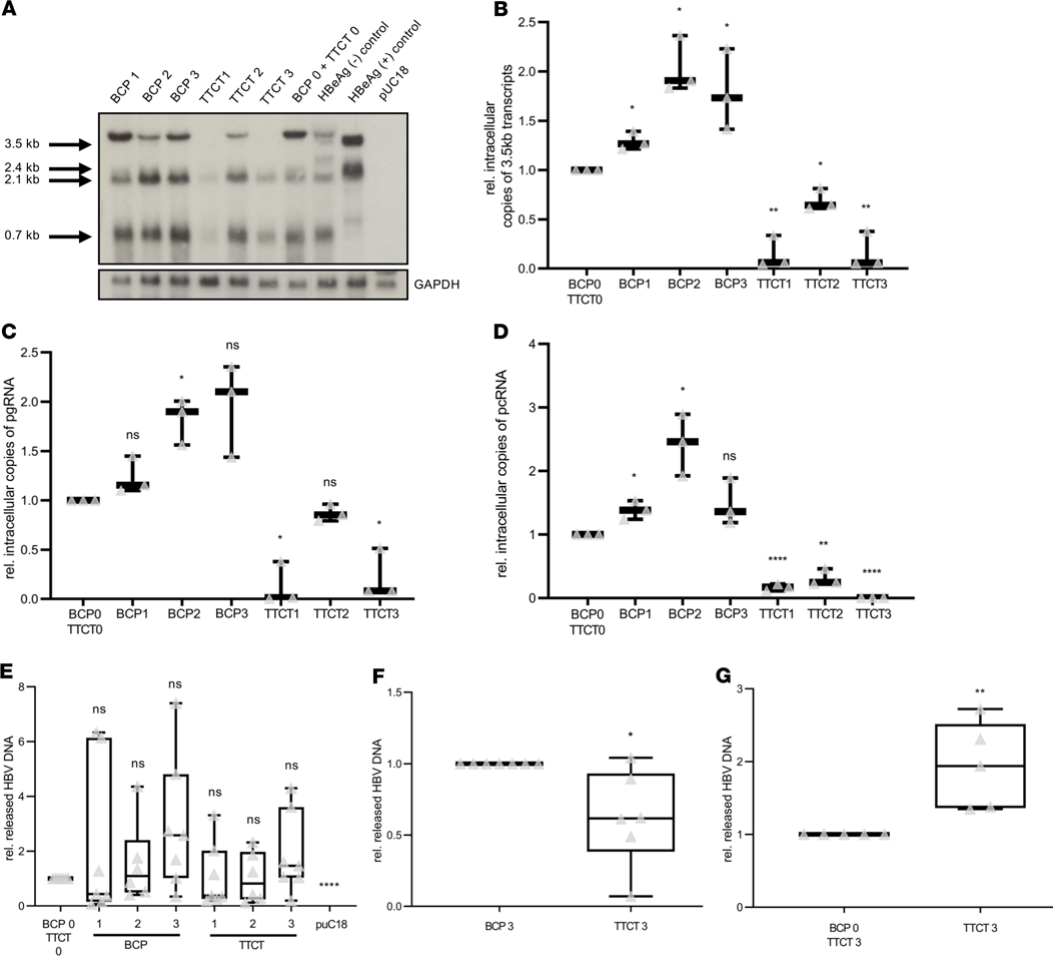
Impaired viral replication by GCAC1809-1812TTCT in vitro. (**A**) Northern blot analysis; as additional control, an HBeAg-negative genotype A genome harboring precore double mutation G1896A/G1899A was used. (**B–D**) Real-time PCR analyses of lysates for total 3.5 kb (**B**), pregenomic (**C**), or precore (**D**) transcripts. (**E–G**) DNA real-time PCR analyses of supernatants. (**G**) An additional an additional genome harboring GCAC1809-1812TTCT in HBx and core promoter but without the A1762T/G1764A BCP double mutation was used (BCP0/TTCT3). Values were normalized to BCP0/TTCT0 (**B–E**) and represent a total value of *n* = 3 (**B–D**), *n* = 7 (**E** and **F**), and *n* = 5 (**G**) independent experiments. In **F**, values of **E** were normalized to BCP3 and in **G** values were normalized to TTCT3. Data are shown as follows (**B–G**): median (line inside the box); first and third quartile (upper and lower limit of the box, respectively); and the highest and lowest values are represented by the top and bottom whiskers. Multiple *t* test with the Holm-Šidák method was performed to correct for multiple group comparisons and to determine statistical significance in **B**–**E**, a 2-tailed student *t* test was performed to determine statistical significance in **F** and **G**. *P < 0.05, **P < 0.01, ***P < 0.001, ****P < 0.0001. HBsAg, HBV surface antigen; BCP, basal core promoter.

To further investigate if GCAC1809-1812TTCT might influence the synthesis of the different HBV-specific transcripts, we performed Northern blot analysis. The Northern blot revealed that all HBV transcripts were detectable in all variants (Figure 4A). However, besides reduced *HBx*-specific (0.7 kb) and *HBsAg*-specific (2.1 and 2.4 kb) transcripts, markedly less 3.5 kb RNA transcripts were detected in the case of the 2 variants harboring GCAC1809-1812TTCT in the core promoter position (TTCT1 and TTCT3). In the case of the other constructs, all RNA transcripts were detected in almost comparable amounts. In line with the Northern blot, 3.5 kb transcripts measured by real-time PCR were also drastically reduced in the TTCT1 and TTCT3 variants (Figure 4B). Here, a moderate reduction of 3.5 kb transcripts was also observed in the TTCT2 construct with the mutation in the HBx position. In contrast, in cells expressing BCP double mutation either in the promoter or the HBx position, the amount of 3.5 kb transcripts was moderately increased. For further differentiation of the 3.5 kb transcripts in pregenomic(pg)RNA and precore mRNA, real-time PCR was also used. We observed that although GCAC1809-1812TTCT in both positions (TTCT1–3) led to a strong reduction of precore mRNA, a reduction of pgRNA was observed only in the variants with this mutation in the promoter position (Figure 4, C and D). In contrast, the amount of pgRNA and precore mRNA was moderately increased by all constructs harboring only the BCP double mutant.

Real-time PCR analyses of supernatants (Figure 4E) detected no significant differences among all variants in comparison with the variants without any of these mutations (BCP0-TTCT0). Because BCP double mutation was described in some studies to increase pgRNA encapsidation and all of our analyzed GCAC1809-1812TTCT constructs (TTCT1–3) harbor the additional BCP double mutation (BCP3), we analyzed if the addition of GCAC1809-1812TTCT in the BCP variant affects HBV DNA release. Here, we observed that when GCAC1809-1812TTCT was present in both positions, extracellular HBV DNA levels were slightly but significantly decreased in comparison with the BCP3 variant (Figure 4F). For further analyses, we included another construct harboring the GCAC1809-1812TTCT mutation (BCP0-TTCT3) without the additional BCP double mutation and compared it with the TTCT3 variant. Here, we observed in the GCAC1809-1812TTCT variant without the BCP mutation a significantly diminished release of HBV DNA of approximately 2-fold in comparison with the variants that harbor the additional BCP double mutation (Figure 4G).

### Changes in thermodynamic stability of pgRNA secondary structures in the context of BCP and GCAC1809-1812TTCT mutations.

**Figure 5:**
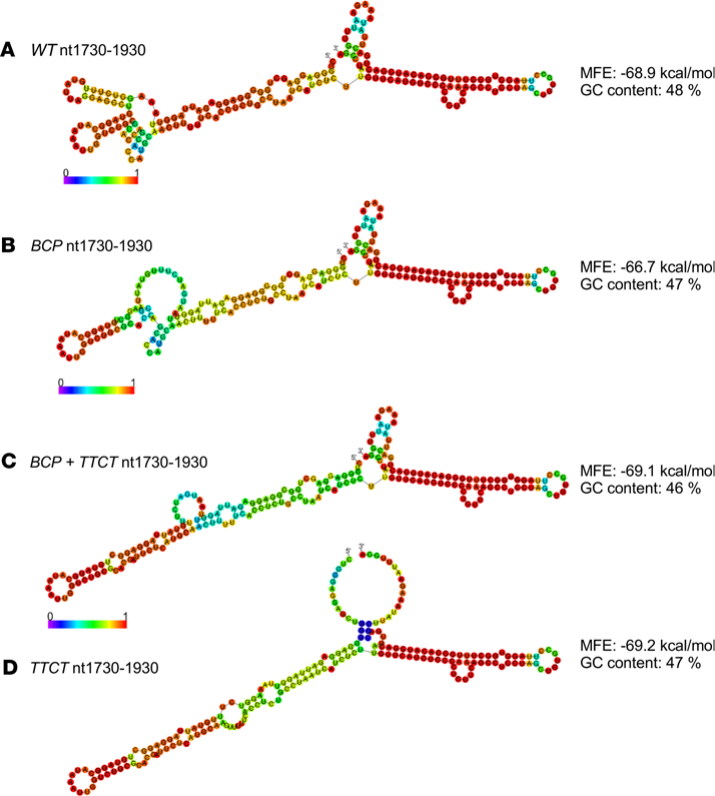
Thermodynamic destabilization of the pgRNA secondary structure by A1762T/G1764A and compensation by GCAC1809-1812TTCT. (**A–D**) Calculation of the thermodynamic stability of nt1730–1930 pgRNA genomic region genome with the presence of (**A**) none of the mutations, (**B**) A1762T/G1764A, (**C**), A1762T/G1764A and GCAC1809-1812TTCT, and (**D**) GCAC1809-1812TTCT without A1762T/G1764A. The used HBV sequence was derived from an inactive carrier patient with the coexistence of A1762T/G1764A and GCAC1809-1812TTCT. Red bars indicate the positions in the sequences where the mutations are localized. BCP, basal core promoter; MFE, minimum free energy; GC, guanine–cytosine.

As described above, GCAC1809-1812TTCT is highly prevalent in inactive carriers and strongly associated with BCP double mutation A1762T/G1764A. Furthermore, this mutation leads to lower HBV DNA levels in vivo and negatively impacts the synthesis of pgRNA and viral proteins in vitro. To analyze if the coexistence of the BCP and GCAC1809-1812TTCT mutations might influence HBV pgRNA secondary structure, calculation of the thermodynamic stability of nt1730–1930 pgRNA genomic region was performed for these 2 mutations as recently described (17). We calculated that the RNA secondary structure of the A1762T/G1764A containing pgRNA is thermodynamically less stable with an increase in the minimum free energy (MFE) value of 2.2 kcal/mol compared with the WT pgRNA secondary structure (Figure 5, A and B). The calculated MFE of the A1762T/G1764A containing pgRNA appeared to be higher (–66.7 kcal/mol; GC content of 47%) in comparison with pgRNA lacking this mutation (–68.9 kcal/mol; GC content of 48%). After the implementation of GCAC1809-1812TTCT in addition to A1762T/G1764A (Figure 5C), the RNA secondary structure was stabilized by a hairpin structure and the MFE decreased approximately to the WT level (–69.1 kcal/mol; GC content of 46%). However, the introduction of GCAC1809-1812TTCT without A1762T/G1764A (Figure 5D) resulted in a calculated MFE (–69.2 kcal/mol; GC content of 47%), which is comparable to the thermodynamic stability of the WT sequence. These data suggest that while the introduction of A1762T/G1764A leads to a slight destabilization of the pgRNA secondary structure, GCAC1809-1812TTCT might restore the thermodynamic stability of the pgRNA.

### GCAC1809-1812TTCT was absent in patients with HBV-related compensated liver cirrhosis.

**Figure 6:**
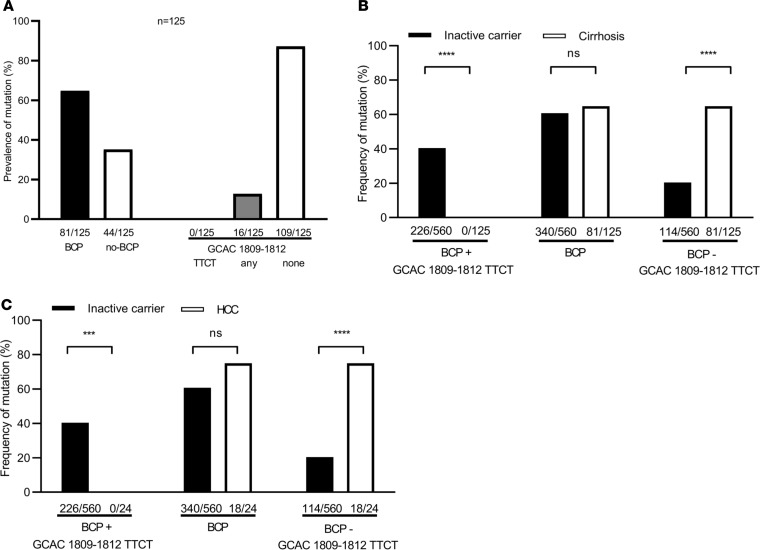
GCAC1809-1812TTCT is absent in patients with cirrhosis and strongly associated with inactive carrier status. (**A**) Prevalence of BCP double mutation A1762T/G1764A and mutations at nt1809–1812 in patients with liver cirrhosis. (**B** and **C**) Association of A1762T/G1764A with or without GCAC1809-1812TTCT with (**B**) the presence of liver cirrhosis and (**C**) the presence of liver cirrhosis and HCC in comparison with inactive carriers. A χ^2^ test was used for group comparisons. *P < 0.05, **P < 0.01, ***P < 0.001, ****P < 0.0001. BCP, basal core promoter.

To analyze the prevalence of GCAC1809-1812TTCT in patients with advanced HBV-related liver disease, sequencing of the BCP region was performed in viremic serum samples from another European cohort of 125 patients with HBV-related compensated liver cirrhosis, including 24 patients with history/presence of HCC or development of HCC after sampling (patient demographics are summarized in Table 2). The main GTs were GTA and GTD in this population (10.4% and 74.4%, respectively). Overall, the A1762T/G1764A mutation was found in the vast majority of samples (65%, 81 of 125). However, although any other mutation (single, double, or triple point mutation) in the Kozak sequence nt1809–1812 was detected in 15% (16 of 125) of the samples, the quadruple mutation GCAC1809-1812TTCT was not detected in a single sample (0%, 0 of 125) (Figure 6A).

### GCAC1809-1812TTCT acted as a biomarker.

To examine if GCAC1809-1812TTCT might act as a potential biomarker, its prevalence was compared among the different cohorts. The prevalence of GCAC1809-1812TTCT was significantly higher (*P* < 0.0001) in our cohort of inactive carriers in comparison with patients with established compensated liver cirrhosis (Figure 6B). Although GCAC1809-1812TTCT in combination with BCP was found in 40% of the inactive carriers, this combination was absent in patients with cirrhosis. Additionally, a strong association with inactive carrier status was found when compared with the subgroup of patients with established compensated liver cirrhosis and HCC (Figure 6C).

Next, we asked if the GCAC1809-1812TTCT status might be able to enhance the specificity of BCP double mutation as a prognostic marker for advanced HBV-related liver disease. We observed that the presence of BCP alone without further specification of GCAC1809-1812TTCT status was associated with neither inactive carrier status nor established compensated liver cirrhosis or HCC (Figure 6, B and C). In contrast, the presence of BCP double mutation without coexistence of GCAC1809-1812TTCT was strongly associated with liver cirrhosis and HCC. Although BCP double mutation without GCAC1809-1812TTCT was found only in 20% (114 of 560) of the inactive carrier patients, it was found in the majority of patients with compensated liver cirrhosis (65%, 81 of 125) and the HCC subgroup (75%, 18 of 24).

## Discussion

Recently, we performed extensive mutation analyses of the core, precore, and preS regions in a large European cohort of inactive HBV carriers (16). In the present study, 560 patients from this cohort were analyzed for additional core mutations associated with BCP double mutation A1762T/G1764A. As recently described, the BCP double mutation A1762T/G1764A is frequently (61%) found in the overall study population (16, 18). In addition to the frequently described BCP double mutation, in this study we describe a highly prevalent and prognostic relevant quadruple point mutation (GCAC1809-1812TTCT) in the Kozak sequence directly preceding the precore initiation codon. Located directly between 2 highly conserved regions, which are involved in the regulation of transcription (nt1770–1808 and nt1813–1849) (19), this mutation was found in 42% of the overall study population and frequently among all major HBV GTs (GTA–GTE). It leads to the substitution of alanine to phenylalanine and proline to serine in the region around the precore initiation codon (nt1808–1817), which is highly conserved in all GTs (20). In addition, due to the overlapping open reading frames of the HBV genome, this mutation also leads to corresponding changes in the downstream region of the HBx gene. Different double and triple point mutations at nt1809–1812 were described in 2 studies from South Africa, including patients infected with HBV GTA (14, 20). The double point mutation G1809T/C1812T was observed to reduce HBeAg synthesis in vitro (20). In a German study, different point mutations at nt1809–1812 were recently described in patients with HIV/HBV coinfection, HBV blood donors, and patients with HBV-related chronic liver disease (21).

In our study, we observed a strong association of GCAC1809-1812TTCT with BCP double mutation A1762T/G1764A in the inactive carrier group. Although the majority of samples with the presence of BCP double mutation was also positive for GCAC1809-1812TTCT, this additional mutation was found only infrequently in samples without BCP double mutation. In addition, GCAC1809-1812TTCT but not BCP double mutation is significantly associated with lower HBV DNA levels. Therefore, our recent, unexpected observation that the BCP double mutation was associated with lower HBV DNA levels (16) can be explained by the high prevalence of GCAC1809-1812TTCT in patients with BCP double mutation.

The impact of BCP double mutation on molecular virology was studied in several other in vitro and in vivo studies (16, 19, 22–30) with heterogeneous results. Although precore RNA was found to be decreased in most studies, mostly minor impacts on levels of pgRNA and no significant impact on HBsAg-specific RNAs were observed in most of these in vitro studies. In addition, an increase of pgRNA encapsidation and synthesis of progeny virus was observed by some groups. Interestingly, BCP double mutation was found in both HBeAg-positive and HBeAg-negative patients, underlining that this mutation does not completely abolish HBeAg, like, for example, the precore mutation G1896A, which converts TGG to the stop codon TAG (18). In our study, the amount of 3.5 kb transcripts was moderately increased in cells expressing BCP double mutation in either the promoter or the HBx position, which is in line with several publications (19, 28). But, interestingly, in addition to the pgRNA, the precore mRNA was also moderately increased by all variants harboring the BCP double mutant, including the variant with this mutation in the HBx position (BCP). In contrast to our data, decreased levels of precore mRNA were found in some studies (23, 24, 28). Nevertheless, in other studies, only low or moderate changes in precore mRNA and HBeAg release were observed (25, 29). As demonstrated by Parekh et al., the impact of BCP double mutations and other mutations strongly depends on the genetic background of the isolates (29). In line with other studies, extracellular HBV DNA was not significantly altered (24, 25, 30).

In addition, we analyzed the impact of GCAC1809-1812TTCT on viral transcripts, HBsAg/core expression, and HBeAg/HBV DNA release. We found that GCAC1809-1812TTCT drastically reduced the amount of both 3.5 kb transcripts and to a lesser extent the level of the other viral transcripts (2.4/2.1/0.7 kb). This was observed only when the mutation was present in the core promoter position, but not when it was present in the HBx position, which indicates a promoter-mediated HBx-independent mechanism. The observed reduction of viral transcripts leads to reduced synthesis of HBsAg, core, and HBeAg, as detected in our in vitro study. However, why these mutations, which are located in the promoter of the core gene, also affect transcription of HBs and HBx RNAs remains unclear. Because cccDNA synthesis can also be induced in hepatoma cells (31), one possible explanation might be that due to the reduced transcription of pgRNA, less pgRNA might be available to restore the cccDNA pool, which might subsequently reduce the overall transcriptional activity. In addition, GCAC1809-1812TTC may also affect the structure of the DNA template, which might again reduce transcription of all transcripts. However, further research has to be performed to address this question. Although we observed that GCAC1809-1812TTCT reduced HBsAg synthesis in vitro, interestingly, no differences in HBsAg levels were detectable in our patients. This can be explained by several recent reports including one from our group, in which patients’ sera of the Albatros study were also analyzed. In these studies, it was concluded that integrated DNA has to be considered as a potent source for sufficient HBsAg expression in HBeAg-negative patients (32–34). Therefore, integrates might also contribute to compensation of an impaired HBsAg secretion in our patients.

Extracellular HBV DNA levels were not significantly changed by GCAC1809-1812TTCT in our in vitro study when compared with our reference genome, although pgRNA levels were strongly decreased and lower serum HBV DNA levels were observed in our patients’ sera. BCP double mutation was described in some studies to increase pgRNA encapsidation and synthesis of progeny viruses in vitro. As all our analyzed GCAC1809-1812TTCT variants harbor the additional BCP double mutation, a diminishing effect of the GCAC1809-1812TTCT mutation on virion release might be curtained by the additional presence of the BCP double mutation. Indeed, we observed that the presence of GCAC1809-1812TTCT without the BCP double mutation leads to significantly lower levels of extracellular HBV DNA, although this effect is only moderate and other factors have to be considered. In our patients, lower pgRNA levels due to GCAC1809-1812TTCT might negatively impact the capacity for restorations of the cccDNA pool (19). This might lead to a reduction of transcriptional active cccDNA over a longer period of time and subsequently to a reduction of serum HBV DNA levels. In addition, the expression of core protein is reduced by GCAC1809-1812TTCT, which is required for virion synthesis.

Next, we addressed the question of why GCAC1809-1812TTCT was found so frequently in inactive carriers. In one study, evidence was gained that core promoter mutations might arise as a result of RNA secondary structural considerations and that BCP double mutation leads to significant changes in the secondary pgRNA structure (17). To investigate whether GCAC1809-1812TTCT might act as a compensatory mutation for BCP double mutation, we conducted analog bioinformatical modeling of the secondary pgRNA structure of this region. Interestingly, the secondary pgRNA structure including the BCP double mutation was calculated to be less thermodynamically stable in comparison with the WT sequence. However, after the additional introduction of GCAC1809-1812TTCT, the thermodynamic stability of the pgRNA secondary structure was restored approximately to the WT level. In summary, in the case of the BCP double mutation, the thermodynamic stability of the pgRNA seems to be decreased in favor of an increased replication capacity, which might be beneficial for the virus in stages of low immune pressure for example in the immune tolerant phase. However, in later stages of the disease when the evolutionary pressure again increases (i.e., in the immune clearance phase), continuous hepatitis activity and hepatic flares might result in declining serum HBV-DNA levels and eventually lead to HBeAg seroconversion and development of anti-HBe (27). At this point, GCAC1809-1812TTCT might offer an evolutionary advantage as a compensatory mutation for BCP as GCAC1809-1812TTCT, in contrast to BCP double mutation, leads to a strong reduction of HBeAg and it enhances the thermodynamic stability of the secondary structure of the pgRNA by a local energy minimum, which impairs unfolding of the RNA and might therefore lead to a lower translation and reverse transcription. These mechanisms of enhanced stability, lower protein synthesis, and, importantly, HBeAg reduction mediated by GCAC1809-1812TTCT may act as an escape mechanism of the virus.

To investigate whether GCAC1809-1812TTCT might also act as a biomarker with potential prognostic value, we analyzed another cohort of 125 patients with compensated HBV-related liver cirrhosis for the existence of this mutation. Importantly, although other than GCAC1809-1812TTCT mutations at nt1809–1812 were present in 15% of the patients, which is in line with another study (21), the quadruple mutation GCAC1809-1812TTCT was not found in this cohort at all. Therefore, GCAC1809-1812TTCT was found to be strongly associated with inactive carrier status, and therefore a benign course of the disease in our study. Although described as a marker for advanced HBV-related liver disease, the overall prevalence of BCP double mutation was not found to be associated with the presence of compensated liver cirrhosis or HCC. But, very interestingly, the presence of BCP double mutation alone without additional GCAC1809-1812TTCT mutation was found to be strongly associated with established compensated liver cirrhosis and HCC.

Therefore, we suggest that the GCAC1809-1812TTCT mutation is a promising and robust biomarker for a benign course of the disease. In addition, this mutation might also have the potential to increase the specificity of BCP double mutation as a marker for an unfavorable prognosis. Hence, GCAC1809-1812TTCT should be evaluated in more detail in further studies for possible clinical use. As a limitation of our study, mostly patients infected with HBV GTA and GTD were included, especially in the cirrhotic cohort, and it is uncertain if the results can be generalized for all HBV GTs. Therefore, studies of infected patients with a higher percentage of other HBV GTs (especially GTB and GTC) are needed. In addition, further studies should include precirrhotic patients with HBV-related hepatitis and more patients with HBV-related HCC to evaluate the impact of GCAC1809-1812TTCT as a biomarker in these subgroups of patients.

In conclusion, GCAC1809-1812TTCT, a quadruple mutation in the Kozak sequence preceding the precore start codon, is highly prevalent and strongly associated with BCP double mutation in a large European cohort of inactive carriers. In vitro GCAC1809-1812TTCT leads to drastically diminished HBeAg levels and a diminished replicative activity, which might explain HBeAg negativity and lower HBV DNA levels, as observed in inactive carriers. Importantly, GCAC1809-1812TTCT was not found at all in a large cohort of patients with compensated liver cirrhosis, suggesting this mutation is a potential biomarker for HBV-related disease.

## Methods

### Study populations.

A total of 560 participants of the German Albatros trial (NCT01090531) with inactive carriers were included in the analysis. For main inclusion and exclusion criteria of the Albatros trial, see the Supplemental Methods. The serum or plasma of these patients was prospectively collected and stored at –80°C. Analyses of HBV DNA viral load and qHBsAg were limited to patients with available HBV DNA and qHBsAg levels determined in clinical routine (*n* = 560 [100%] and *n* = 524 [92%], respectively). In addition, serum samples of 125 patients with HBV-related compensated liver cirrhosis including patients with history/presence or development of HCC after sampling were analyzed. HBV GTs were determined either in clinical routine or by direct sequencing of the polymerase region.

### HBV DNA extraction.

Viral DNA was extracted from 200 μl of serum using the QIAamp DNA Blood Mini Kit (QIAGEN) according to the manufacturer’s protocol.

### Primers.

Primers are described in the Supplemental Methods.

### Amplification of the HBV BCP region and full-length HBV genome.

A fragment of the region encoding the BCP gene (nt1681–2044) was amplified by (semi-)nested PCR. The detailed conditions are given in the Supplemental Methods. The entire HBV genome was amplified by using primers P1 and P2 modified from Günther et al. (35) and by using the Expand High Fidelity PCR Kit (Roche).

### Direct sequencing of BCP region and full-length genome.

The corresponding DNA was subjected to sequencing PCR according to the manufacturer’s protocol (BigDyeDeoxy Terminators, Applied Biosystems). The DNA was sequenced on a 3130xl Genetic Analyzer (Applied Biosystems) and a sensitivity level of about 15%–20% was assumed.

### Cell culture and cell treatment.

The human hepatoma-derived cell line Huh 7 was cultivated as previously described (36).

### Plasmids.

HBV genomes containing A1762T/G1764A and GCAC1809-1812TTCT in different positions in the core promoter and HBx genomic region were commercially synthesized (GenScript; for an overview of analyzed HBV genomes, see Figure 2A). For control of the assays, a GTA HBeAg-negative genome with precore double mutation G1896A/G1899A (16), a GTA HBeAg-positive genome (37). and pUC18 vector were used.

### Chemicals, antibodies, and enzymes.

Chemicals, antibodies, and enzymes used for Western blotting and immunofluorescence microscopy are described in see Supplemental Methods. HBsAg-specific (Enzygnost, Siemens), HBeAg-specific (Cusabio), and HBcAg-specific (Cell Biolabs) ELISA were used according to the manufacturer’s protocol.

### SDS-PAGE and Western blot analysis.

Protein was extracted using RIPA followed by a sonication step for 10 seconds. SDS-PAGE and Western blot analysis were performed according to standard procedures (38).

### Northern blot analysis.

RNA was isolated using Trizol. Northern blot analysis was performed according to standard procedures (38).

### Transfection of cells.

For transient transfection, Huh 7 cells were transfected using 6 μl/μg DNA polyethyleneimine or X-tremeGENE DNA Transfection Reagent with a ratio of 3:1 of the reagent to μg plasmid DNA (Polysciences) according to the manufacturer`s instructions. Cells and supernatants were harvested 48 hours after transfection and stored at –20°C. Transfection efficiency was determined by immunofluorescence analysis based on the number of positive cells using an HBsAg-specific antibody (HB01, see Supplemental Methods), as no major differences were notable and no corrections were made.

### RNA isolation and cDNA synthesis.

Frozen cells were lysed using the TriFast reagent (Peqlab) and the RNA isolated by phenol/chloroform extraction. Extracted RNA was solubilized in 15 μL DEPC-H_2_O and 4 μg RNA were used for subsequent cDNA synthesis. All samples were incubated with DNase I at 37°C for 1 hour and subsequently incubated with a random hexamer primer to begin cDNA synthesis. Samples were incubated at 42°C with a Mastermix containing 10 Mm dNTPs, reverse transcriptase (RT), and corresponding buffer (Thermo Fisher). Finally, the RT was inactivated at 72°C for 10 minutes. cDNA samples were diluted 1:10 and directly used for real-time PCR.

### Indirect immunofluorescence analysis.

Immunofluorescence staining was performed as previously described (39) and analyzed using a confocal laser scanning microscope and ZEN 2012 software (Carl Zeiss).

### Calculation of pregenomic RNA secondary structures of nt1730–1930 genomic region of HBV.

To predict the secondary structures of the pgRNA regions and for calculation of the thermodynamic stability of the folded RNAs, the web service *RNAfold* (40) was used. nt1730–1930 as well as the same RNA region containing the respective mutations were entered and the MFE was calculated, avoiding isolated base pairs and therefore helices of a length of 1.

### Statistics.

χ^2^ Test or Fisher’s exact test was used for group comparisons of categorical data and the Wilcoxon-Mann-Whitney *U* test was used for group comparisons of ordered data as appropriate. Differences within HBV DNA and qHBsAg serum levels were determined using the nonparametric Kruskal-Wallis test with post hoc Dunn’s test. Single nonparametric 2-tailed *t* tests were used for analyses of in vitro experiments with 2 groups and multiple 2-tailed *t* test with the Holm-Šidák method were performed to correct for multiple group comparisons and to determine statistical significance for analyses of the in vitro experiments with more than 2 groups. Outliers were identified using the ROUT method (Q = 1%). Statistical analysis was done with BiAS for Windows, version 11 (Epsilon) and GraphPad Prism software version 8. A *P* value of less than 0.05 was considered significant.

### Study approval.

The study was approved by the local ethic committee of University Clinic of Frankfurt (97/09), and written informed consent was obtained from all patients. It was not appropriate or possible to involve patients or the public in the design, conduct, reporting, or dissemination of our research. The study was performed in accordance with the provisions of the Declaration of Helsinki and good clinical practice guidelines.

## Author contributions

KHP was guarantor of the article. KHP and E. Hildt conceived and designed the study. JV, VK, JD, FF, JT, PL, CG, and C. Sarrazin acquired the data. KHP, E. Hildt, C. Spengler, MB, BJ, MG, LK, WO, TZ, GC, AK, and AG analyzed and interpreted the data. KHP, C. Spengler, MB, AG, and E. Hildt drafted the manuscript. SZ, C. Sarrazin, and PL provided critical revision of the manuscript for important intellectual content. MB, MG, C. Spengler, and E. Herrmann provided statistical analysis.

## Supplementary Material

supplemental data

## Figures and Tables

**Table 1:**
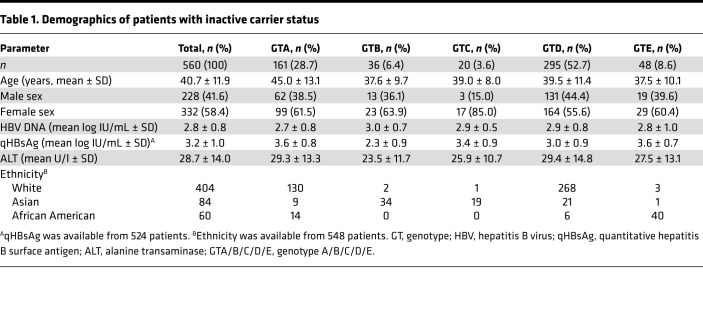
Demographics of patients with inactive carrier status

**Table 2:**
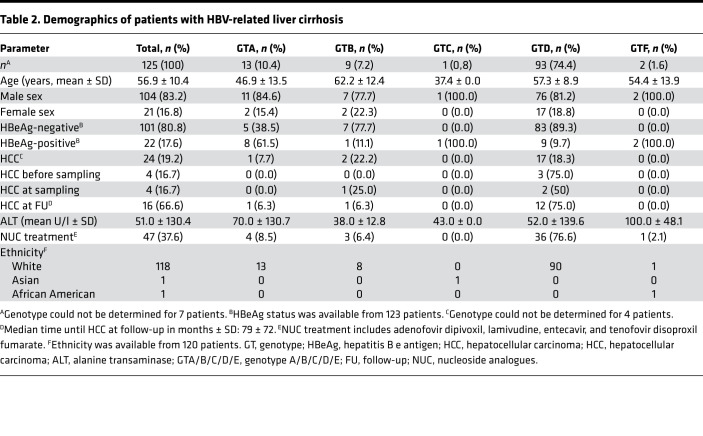
Demographics of patients with HBV-related liver cirrhosis

## Version 1 10/15/2020

In-Press Preview

## Version 2 11/19/2020

Electronic publication
