# Supplementary material for: Quadruple mutation GCAC1809-1812TTCT acts as a biomarker in healthy European HBV carriers
Source: JCI Insight. 2020 Nov 19;5(22):e135833. doi: 10.1172/jci.insight.135833 (PMC7710305; doi:10.1172/jci.insight.135833)
Supplement: supplemental data [file jciinsight-5-135833-s078.pdf]

Supplementary Methods

Main inclusion criteria of the German multicenter trial ALBATROS

- Chronical HBV infection with HBsAg and anti-HBc positivity for at least 6 month
- HBV-DNA < 100,000 IU/ml.
- ALT ≤ 2.0-fold ULN
- Age between 18 and 79

Main exclusion criteria of the German multicenter trial ALBATROS

- Current or intended antiviral therapy against HBV
- Previous antiviral HBV therapy for more than 3 months
- Chronic "Immuntolerant" HBeAg positive HBV infection (HBeAg positive, high levels of HBV-DNA, normal liver values)
- Coinfection with HCV and HIV as well as malignant or psychiatric comorbidities
- Hepatocellular carcinoma or hepatic metastases

Primers

Table 1: Oligonucleotides for PCR, direct sequencing

Oligoname	Sequenz 5' - 3'	Function
1_f	CACGTTGCATGGAGACCA	nested PCR BCP/PC genotype A, D, E
1_r	GGAGTGCGAATCCACACTCC	nested PCR BCP/PC genotype A, D, E
2_f	TGTCAACGACCGACCTTGAG	nested PCR BCP/PC genotype A, D, E; sequencing
2_r	GCAATGCTCAGGAGACTCTAAGGC	nested PCR BCP/PC genotype A, D, E
3_f	ACTCTTGGACTYTCAGCAATG	nested PCR BCP/PC genotype B, C
3_r	GTCAGAAGGCAAAAAAGAGAG	nested PCR BCP/PC genotype B, C
4_f	TCTCAGCAATGTCAACGACCG	nested PCR BCP/PC genotype B, C; sequencing
4_r	AGAGAGTAACTCCACAGAWGCTC	nested PCR BCP/PC genotype B, C
7_f	TGCGGCGTTTTATCATCTTCCT	nested PCR polymerase
7_r	GTTTAAATGTATACCCAAAGAC	nested PCR polymerase
8_f	CAGCGGCATAAAGGGACTCAAG	nested PCR polymerase; sequencing
A1	CCCAAGCTTCTATTGATTGGAAAGTATGTC	Amplification of fragment A
A2	GAAAATTGAGAGAAGTCCAC	Amplification of fragment A
B1	ACAARAATCCTCACAATACC	Amplification of fragment B
B2	GAAGATCTGATAGGGGCATTTGGTGGTC	Amplification of fragment B
K1	TGCCTCTCACATCTCGTCAATC	sequencing full-length genome
K2	GAGCCAAGAGAAACGGACTG	sequencing full-length genome
K3	TCCAGGAACAACAACAAC	sequencing full-length genome
K4	GTTGGCGAGAAAGTGAAAGC	sequencing full-length genome
K5	GGCTTTGCTGCTCCATTTAC	sequencing full-length genome
K6	TTGCTGGGAGTCCAAGAGTC	sequencing full-length genome
K7	CCGTCTGTGCCTTCTCATC	sequencing full-length genome
K8	CTAGATCCCTGGATGCTG	sequencing full-length genome
K9	TCACCATACAGCACTCAG	sequencing full-length genome
K10	GTTTCCCTCTTATATAGAATCC	sequencing full-length genome
K11	CAATTTGTGGGCCCTCTC	sequencing full-length genome
K12	TTAGAGGTGGAGAGATGG	sequencing full-length genome
K13	CAGGCTCAGGGCATATTG	sequencing full-length genome
K14	AGCAGCAGGATGAAGAGG	sequencing full-length genome
HBV_1803f	GCACCAGCACCATGCAACTT	pre-core RNA specific Primer
HBV_1825f	TCACCTCTGCCTAATCATCTCTTG	pre-genomic RNA specific Primer
HBV_1965r	TCAGTAGGCAAAAACGAGAGTAACT	3.5 kb specific reverse Primer

Amplification of the HBV BCP/precore region

Parts of the region encoding the BCP and the precore gene were amplified by nested PCR in two rounds. For genotypes A, D and E outer primers 1_f and 1_r and inner primers 2_f and 2_r were used. Outer primers 3_f and 3_r and inner primers 4_f and 4_r were used for genotypes B and C. PCR was done with the QIAGEN Fast Cycling

Kit *(Qiagen, Germany)*. Outer and inner PCR amplification consisted of an initial denaturation step of 95 °C for five minutes, denaturation for 5 seconds 96 °C, annealing 5 seconds 52 °C/55 °C (outer/inner) and an elongation step of 68 °C for 1 minute for 35 cycles, and a final elongation step of 72 °C for 1 minute.

Amplification of the polymerase region

A part of the *polymerase* gene was amplified by semi-nested PCR in two rounds. For the outer PCR primers 7_f and 7_r and for the inner PCR primers 7_f and 8_R were used. The annealing temperatures were 50 °C and 55 °C for the outer and inner PCR, respectively. Cycling conditions were as described above.

Amplification of the full-length HBV genome

The entire HBV genome was amplified with modified primers P1 and P2 from Günther et al., 1995. The Expand High Fidelity PCR Kit (Roche, Germany) was used and the cycling conditions were as followed: initial denaturation at 94 °C for 2 minutes, followed by 40 cycles of denaturation at 94 °C for 40 seconds, primer annealing at 56 °C for 90 seconds, elongation at 68 °C for 3 minutes with an increment of 2 minutes every tenth cycle.

Amplification of 3.5 kilobase transcripts

The 3.5 kilobase transcripts were amplified with primers HBV_1803f and HBV_1965r for the precore RNA and HBV_1825f and HBV_1965r for the pregenomic RNA. The amount of precore and pregenomic RNA was calculated as previously described by [1, 2]. Cycling conditions were as following: initial denaturation at 95 °C for 10 minutes, followed by 45 cycles of denaturation at 95 °C for 15 seconds, primer annealing at 56 °C for 30 seconds, elongation at 72 °C for 30 seconds.

Northern blot analysis

RNA was isolated as described using TRIZOL according to the instructions of the manufacturer. Agarose electrophoresis and blotting was performed using standard protocols [3]. For detection of HBV-specific transcripts a radioactive –labelled HBx-specific probe encompassing 0.5kB was used. Detection of GAPDH-specific transcripts served as loading control.

Western blot analysis and immunofluorescence microscopy

Western blot analyses were performed as described [4],

The monoclonal MA18/7 LHBs-specific antibody and the monoclonal HB01 SHBs-specific antibody were kindly provided by D. Glebe [5] Giessen, Germany. Both were used in a 1:100 dilution for immunofluorescence experiments. The polyclonal rabbit-derived antiserum against denatured HBV core (K46 [6]) was a gift from Reinhild Prange, Department of Medical Microbiology and Hygiene, Johannes Gutenberg-Universität Mainz, Mainz, Germany. For Western blot analysis K46 was used in a 1:5000 dilution. For immunofluorescence microscopy a polyclonal rabbit anti-HBV core serum (B0586) was purchased from Dako, Denmark and used in a 1:200 dilution. Additionally, a monoclonal mouse antibody specific for HBV core dimers (MAB3120, Tokyo Future Style, Inc. [7]) was used in a 1:60 dilution. Anti-β-actin for loading control in western blot analyses was ordered from Sigma-Aldrich (St. Louis, MO) and used in a 1:1000 dilution. For double immunofluorescence staining, a donkey-derived mouse-specific Cy3-labeled secondary antibody from Invitrogen (Darmstadt, Germany) and a donkey-derived rabbit-specific FITC 488 -labeled secondary antibody were used (Jackson Laboratories, Bar Harbor, ME)

1. Laras A, Koskinas J, Hadziyannis SJ. In vivo suppression of precore mRNA synthesis is associated with mutations in the hepatitis B virus core promoter. Virology 2002;295:86-96.
2. Prakash K, Rydell GE, Larsson SB, et al. High serum levels of pregenomic RNA reflect frequently failing reverse transcription in hepatitis B virus particles. Virol J 2018;15:86.
3. Stoler DL, Michael NL. Nucleic acid blotting techniques for virus detection. In Wiedbrauk DL, Farkas DH (eds) Molecular methods for virus detection. Academic Press, San Diego 1995:39-74.
4. Jiang B, Himmelsbach K, Ren H, et al. Subviral Hepatitis B Virus Filaments, like Infectious Viral Particles, Are Released via Multivesicular Bodies. J Virol 2015;90:3330-41.
5. Kucinskaite-Kodze I, Pleckaityte M, Bremer CM, et al. New broadly reactive neutralizing antibodies against hepatitis B virus surface antigen. Virus Res 2016;211:209-21.
6. Rost M, Mann S, Lambert C, et al. Gamma-adaptin, a novel ubiquitin-interacting adaptor, and Nedd4 ubiquitin ligase control hepatitis B virus maturation. J Biol Chem 2006;281:29297-308.
7. Watts NR, Vethanayagam JG, Ferns RB, et al. Molecular basis for the high degree of antigenic cross-reactivity between hepatitis B virus capsids (HBcAg) and dimeric capsid-related protein (HBeAg): insights into the enigmatic nature of the e-antigen. J Mol Biol 2010;398:530-41.

Suppl. Fig. 1: Association of A1762T/G1764A and GCAC1809-1812TTCT with HBV DNA levels among the HBV genotypes A-E

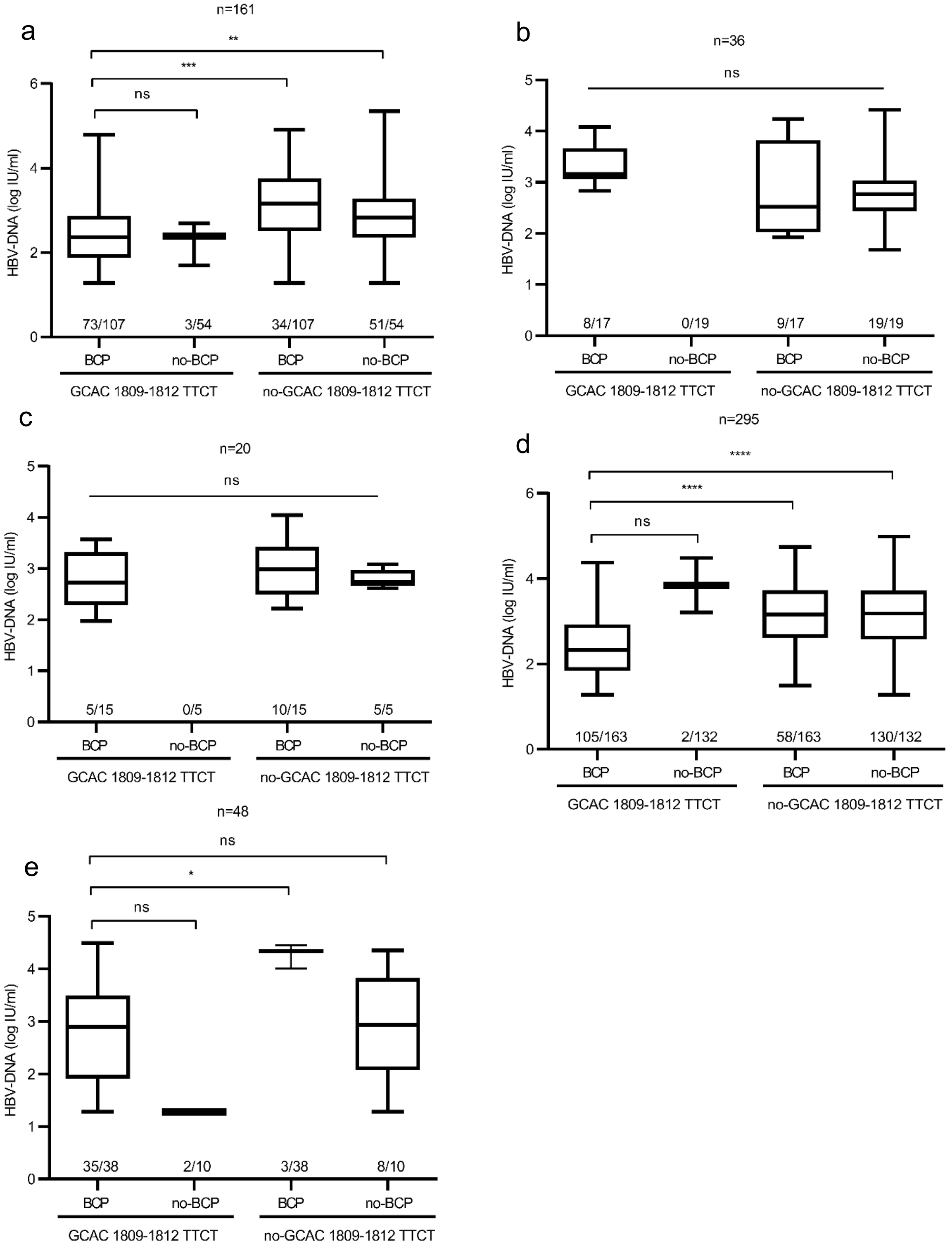

A-E) Association of A1762T/G1764A and GCAC1809-1812TTCT with HBV DNA levels among the HBV genotypes A-E (Figures 1A-E) in inactive carriers; GT, genotypes, Data are shown as following: median (line inside the box), first and third

quartile (upper and lower limit of the box, respectively) and the highest and lowest values are represented by the top and bottom whiskers. A Kruskal-Willis test with post-hoc Dunn’s test was performed to determine statistical significance. *p<0.05, **p<0.01, ***p<0.001, ****p<0.0001.

Suppl. Fig. 2: Association of A1762T/G1764A and GCAC1809-1812TTCT with HBsAg levels among the HBV genotypes A-E

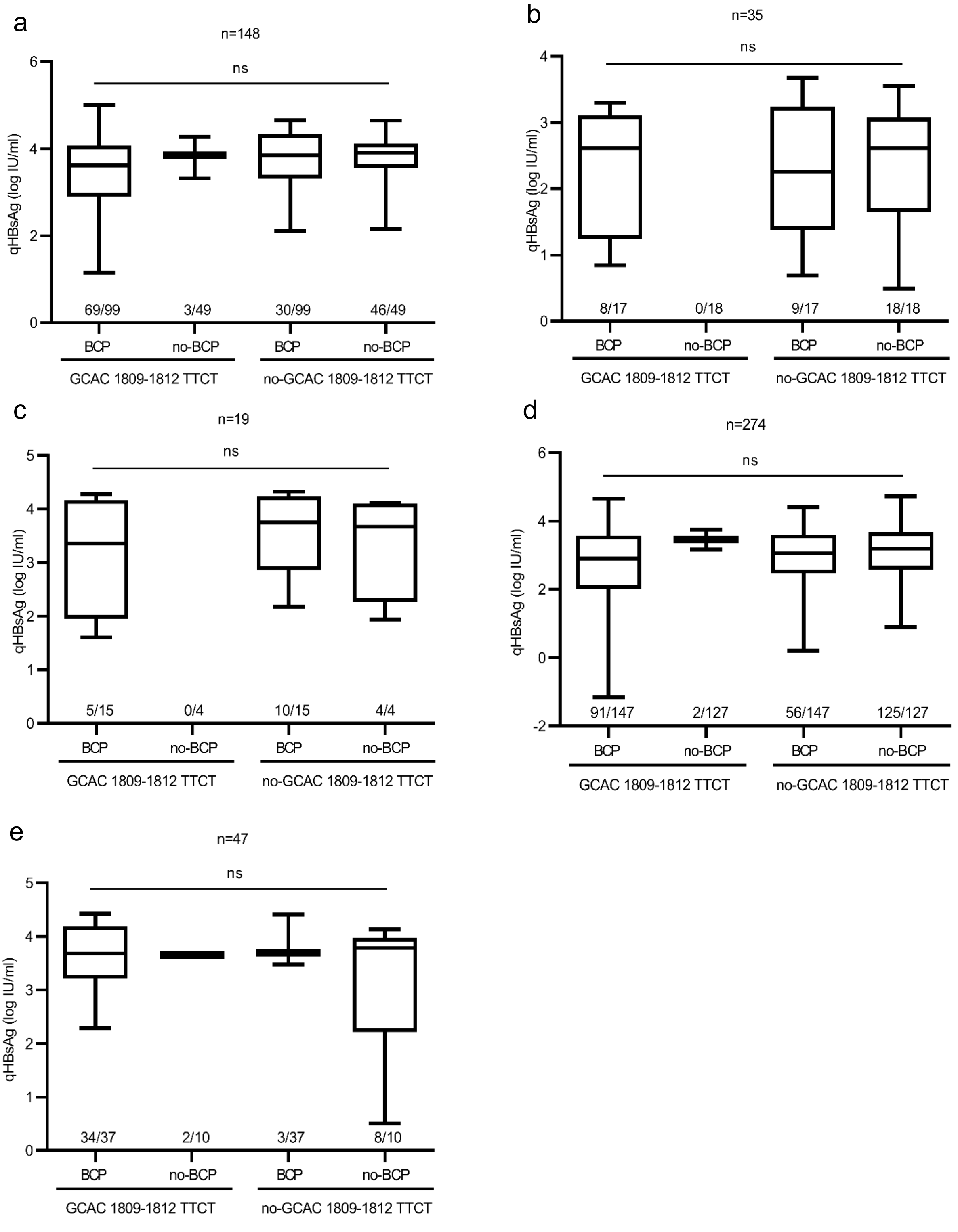

A-E) Association of A1762T/G1764A and GCAC1809-1812TTCT with qHBsAg levels among the HBV genotypes A-E (Figures 2A-E) in inactive carriers; GT, genotypes, Data are shown as following: median (line inside the box), first and third quartile (upper and lower limit of the box, respectively) and the highest and lowest values are represented by the top and bottom whiskers. A Kruskal-Willis test with post-hoc Dunn's test was performed to determine statistical significance. *$p<0.05$, **$p<0.01$, ***$p<0.001$, ****$p<0.0001$.
